# The Mechanism of Ruthenium Oxide Catalyzed Electroless Etching of Silicon in Oxidizing HF Solution

**DOI:** 10.3390/ma19091734

**Published:** 2026-04-24

**Authors:** Bing Bai, Yingqi Li, Wei Xu, Peiao Lu, Jiakun Luo, Jinyu Wu, Kui-Qing Peng

**Affiliations:** Key Laboratory of Multiscale Spin Physics, Ministry of Education, Beijing Key Laboratory of Energy Conversion and Storage Materials, School of Physics and Astronomy, Beijing Normal University, Beijing 100875, China; bingbai@mail.bnu.edu.cn (B.B.); yq_li@mail.bnu.edu.cn (Y.L.); weixu2023@mail.bnu.edu.cn (W.X.); peiaolu@mail.bnu.edu.cn (P.L.); 202521101057@mail.bnu.edu.cn (J.L.); wujy@mail.bnu.edu.cn (J.W.)

**Keywords:** silicon, etching, ruthenium oxide, catalysis

## Abstract

While metal-assisted chemical etching (MACE) or metal-catalyzed electroless etching of silicon in oxidizing HF solutions typically employs noble metals as catalysts, this work investigates oxide-catalyzed chemical etching (OACE) using RuO_2_ to induce localized silicon etching in aqueous H_2_O_2_-HF solutions. RuO_2_ particles confine the reaction to localized sites. The formation of Ru_2_O_3_ during etching suggests that RuO_2_ injects holes into silicon and is simultaneously reduced to Ru_2_O_3_. The oxidized silicon is locally dissolved in aqueous HF solution, and the pores are generated. A cyclic redox mechanism is proposed: RuO_2_ is reduced to Ru_2_O_3_ by extracting electrons from silicon valence band, while Ru_2_O_3_ is rapidly reoxidized by H_2_O_2_, sustaining the etching process until H_2_O_2_ is exhausted. This work challenges the conventional assumption that the catalyst remains unchanged during MACE and offers novel insights into oxide-catalyzed silicon etching mechanisms.

## 1. Introduction

Metal-assisted chemical etching (MACE) is a simple, controllable and cost-effective approach for fabricating a wide range of silicon micro- and nanostructures [1,2,3,4,5,6,7,8]. This method relies on metal catalysts (e.g., Au, Ag, Pt) to induce localized electrochemical reactions during wet etching in HF solutions, enabling selective etching of silicon with nanoscale control over corrosion morphology [9,10,11,12,13]. As demonstrated in previous studies, various desired silicon micro/nanostructures can be obtained by tailoring the catalyst type and morphology, adjusting the composition or concentration of the etchant, modifying the doping type and level of the silicon substrate, or applying additional physical fields (e.g., an external electric field or irradiation) during etching in HF solution [14,15,16,17,18,19,20,21]. From a chemical perspective, an effective MACE should: (i) efficiently catalyze the relevant electrochemical reactions; (ii) exhibit an electronegativity greater than that of silicon; and (iii) be capable of injecting holes into the valence band of silicon, either intrinsically or upon oxidation; in other words, possess a high redox potential [21,22,23,24,25,26,27,28]. Gayrard et al. [29] proposed replacing noble metals with transition metal oxides for this purpose, a process termed oxide-assisted chemical etching (OACE). They suggested that transition metal oxides such as RuO_2_ and IrO_2_ exhibit catalytic properties comparable to those of noble metals. By depositing nanopatterned films of RuO_2_ and IrO_2_ on silicon surfaces and etching in a mixed solution of hydrogen peroxide and hydrofluoric acid, they successfully prepared vertically aligned silicon nanowire arrays. Analogous to metal-catalyzed etching, they attributed this phenomenon to the catalytic injection of holes from hydrogen peroxide into silicon by the metal oxides, thereby inducing the silicon etching reaction. However, although ruthenium oxides are highly effective catalysts for the oxygen evolution reaction (OER), they suffer from limited stability in acidic media [30,31,32,33], and dissolution or corrosion of ruthenium oxides may occur.

In this work, we deposited synthesized RuO_2_ onto silicon surfaces and immersed the samples in a mixed solution of hydrofluoric acid and hydrogen peroxide. During silicon etching, the formation of Ru_2_O_3_ was observed. We propose that RuO_2_ injects holes into silicon and is reduced to Ru_2_O_3_, which is subsequently oxidized to RuO_2_ by hydrogen peroxide. This cycle continues until the hydrogen peroxide in the solution is depleted, at which point the silicon etching ceases. This finding challenges the conventional view that the catalyst is not directly involved in the etching reaction in traditional MACE/OACE models, revealing that transition metal oxides can act as a dynamic ‘hole pump’ during etching, rather than merely serving as an electron transfer medium. Compared with previous studies, we report for the first time the valence state change in RuO_2_ during silicon etching and its synergistic cyclic reaction with silicon and H_2_O_2_.

## 2. Materials and Methods

### 2.1. Materials

One-side polished single crystalline (100)-oriented p-Si wafers (B-doped, ρ = 15–25 Ω·cm) were used in the experiment and purchased from Beijing General Research Institute (Beijing, China) for Non-Ferrous Metals. Ruthenium (III) chloride trihydrate (RuCl_3_·3H_2_O), Hydrogen peroxide (H_2_O_2_), Hydrofluoric acid (HF), sodium hydroxide (NaOH), etc., were purchased from Aladdin Chemical Reagent Co., Ltd., Shanghai (China).

### 2.2. Sample Preparation

Silicon wafers were cleaved into 2 × 2 cm^2^ samples. The cleaved Si wafer pieces were first ultrasonically cleaned in acetone and ethanol for 10 min, respectively, subsequently immersed in a boiling H_2_SO_4_ and H_2_O_2_ (PIRANHA) solution for 30 min. After each cleaning step, the Si pieces were thoroughly rinsed with deionized (DI) water and followed by drying in flowing N_2_.

Synthesis of RuO_2_-1. 0.126 g of RuCl_3_·3H_2_O was dissolved in 100 mL of DI water to prepare a RuCl_3_ solution. Separately, 0.2 g of NaOH was dissolved in 100 mL of DI water to obtain a 0.05 mol/L NaOH solution. Under continuous stirring, the NaOH solution was slowly added dropwise to the RuCl_3_ solution at 30 °C in a water bath until the pH reached 7. The resulting precipitate was collected via vacuum filtration, washed three times with DI water to remove residual ions, and dried in an oven at 60 °C for 12 h. The dried product was ground in an agate mortar and annealed in a tube furnace at 500 °C under a flowing N_2_ atmosphere for 5 h to yield the final RuO_2_-1 sample.

Synthesis of RuO_2_-2. 0.5 g of RuCl_3_·3H_2_O was dissolved in 25 mL of DI water. The solution was sonicated for 30 min and then magnetically stirred for 3 h. The mixture was transferred to a Teflon-lined autoclave and subjected to hydrothermal treatment at 120 °C for 12 h. After cooling, the product was separated by centrifugation and washed three times with DI water, then dried at 80 °C for 72–96 h. The dried material was annealed in a tube furnace at 500 °C under a N_2_ atmosphere for 5 h to obtain the RuO_2_-2 sample.

RuO_2_-catalyzed corrosion of silicon. The as-synthesized RuO_2_ particles were uniformly dispersed onto the surface of a silicon wafer piece. To prevent particle displacement during etching, the coated wafer was covered with another clean silicon wafer of the same size. The stacked wafers were immersed in a H_2_O_2_-HF solution for etching. After etching, the wafer piece was rinsed with DI water and dried with N_2_ gas.

### 2.3. Characterization and Electrochemical Measurements

The morphology of etched silicon surface was examined using scanning electron microscopy (SEM, Gemini 300, ZEISS, Oberkochen, Germany). The chemical states of Ru species were analyzed by X-ray photoelectron spectroscopy (XPS, ESCALAB 250Xi, Thermo Fisher Scientific, Waltham, MA, USA) with an Al Kα X-ray source. The crystal structure was characterized by X-ray diffraction (XRD, D8 Advance, Bruker, Karlsruhe, Germany) with Cu-Κα radiation. The cathodic current density during the corrosion process was measured using a Zennium electrochemical workstation (IM6, Zahner, Kronach, Germany). Elemental composition was analyzed by energy-dispersive X-ray spectroscopy (EDS, Ultim MAX, Oxford, UK).

## 3. Results and Discussion

**Figure 1:**
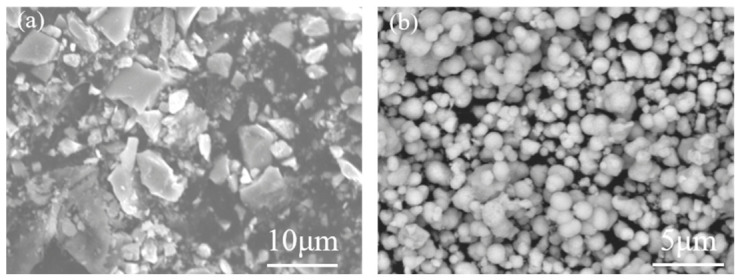
Top-view SEM images of (**a**) RuO_2_-1 and (**b**) RuO_2_-2.

**Figure 2:**
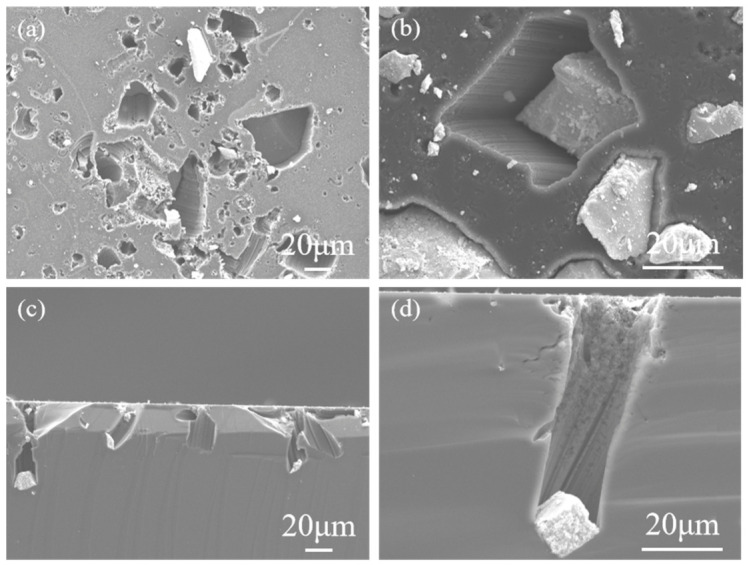
Top-view SEM images (**a**,**b**) and cross-sectional SEM images (**c**,**d**) of RuO_2_-1-coated p-Si (100) 15–25 Ω·cm substrate etched in 0.4 M H_2_O_2_-10 M HF solution for 1 h.

Figure 1 shows the characterization of RuO_2_-1 and RuO_2_-2 particles prepared via Synthesis 1 and Synthesis 2, respectively. Figure 2 presents top-view and cross-sectional SEM images of a RuO_2_-1-coated single-crystalline Si (100) wafer after 1 h of etching in a HF-H_2_O_2_ solution. The top-view images (Figure 2a,b) show that etching occurred exclusively at RuO_2_ particle sites, with pore geometries faithfully replicating the irregular morphology of the catalyst particles. Regions without RuO_2_ remained unetched, confirming that RuO_2_ is essential for initiating corrosion. Smaller particles generated shallow pores, whereas larger ones produced deeper pores. High-magnification SEM (Figure 2b) reveals uneven pore walls, which can be attributed to the rough surface topography of RuO_2_-1 that embeds into the Si substrate and induces localized etching. Cross-sectional analysis (Figure 2c,d) further demonstrates that the etching depth is positively correlated with the particle size. The larger the particle, the greater the corresponding etching depth, whereas smaller particles remain confined near the surface region.

**Figure 3:**
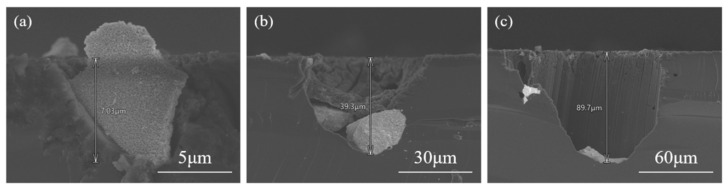
Cross-sectional SEM images of etching depth varying with the size of RuO_2_ particles. The depths of the pores are (**a**) 7.03 μm; (**b**) 39.3 μm; (**c**) 89.7 μm.

As the RuO_2_ particle size increases from approximately 5 μm to 30 μm and further to 60 μm, the etching depth correspondingly increases from 7.03 μm to 39.3 μm and further to 89.7 μm (Figure 3). The size-dependent behavior suggests that more holes are generated in the vicinity of large particles. Notably, the pore diameters near the Si surface were larger than those at the etching front (Figure 2d).

**Figure 4:**
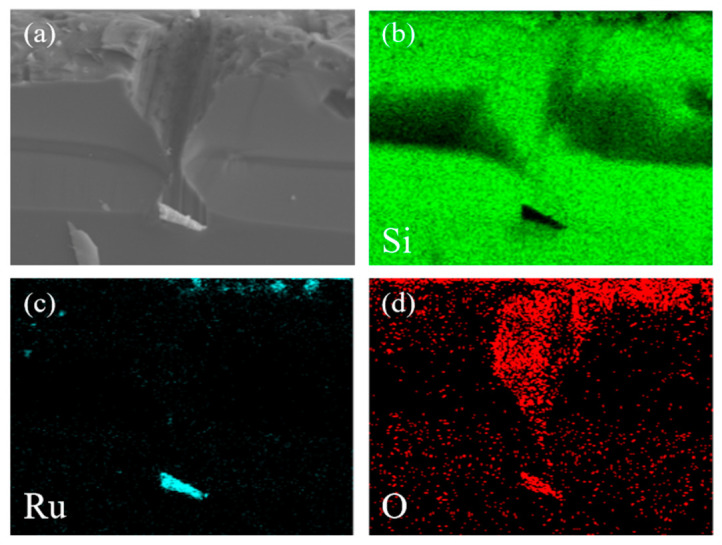
EDS elemental mapping images of Si after RuO_2_-catalyzed etching. (**a**) Cross-sectional SEM image of the sample. Corresponding elemental mapping of (**b**) Si; (**c**) Ru; (**d**) O.

The EDS elemental mapping (Figure 4) clearly shows that selective etching of the Si substrate occurs exclusively at sites where RuO_2_ particles are present. In contrast, regions without RuO_2_ remain unetched, confirming that the localized etching behavior is directly induced by the RuO_2_ catalyst.

**Figure 5:**
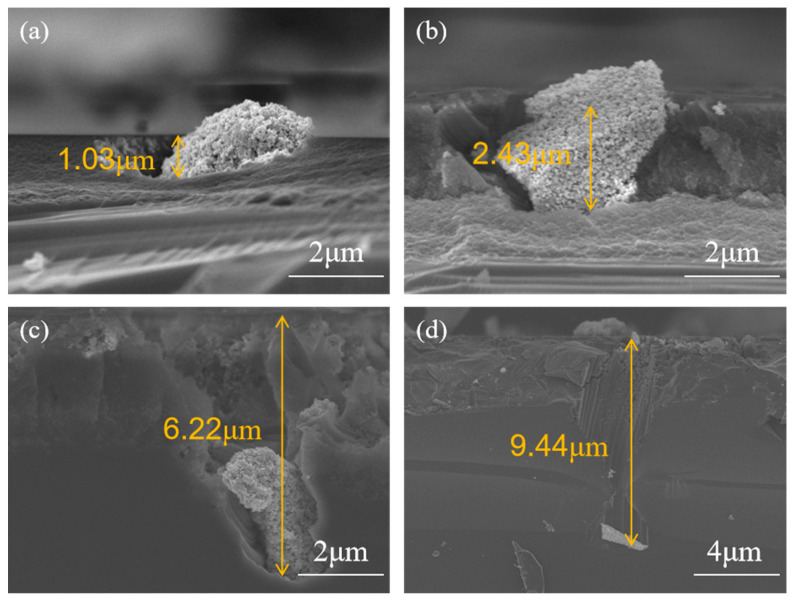
Cross-sectional SEM images of RuO_2_-coated Si etched for (**a**) 10 min; (**b**) 30 min; (**c**) 1 h; (**d**) 2 h.

A quantitative analysis of pore depth with the etching time (Figure 5) was performed using RuO_2_ particles with a diameter of approximately 2 µm. The results show that the etching depth increases with time, reaching 1.03 µm, 2.43 µm, 6.22 µm, and 9.44 µm at 10 min, 30 min, 1 h, and 2 h, respectively. Notably, the evolution of etching depth does not follow a linear relationship with time, indicating a time-dependent decrease in the etching rate. This behavior suggests that the process is kinetically controlled, where the gradual depletion of H_2_O_2_, leads to a progressive deceleration of the etching kinetics.

**Figure 6:**
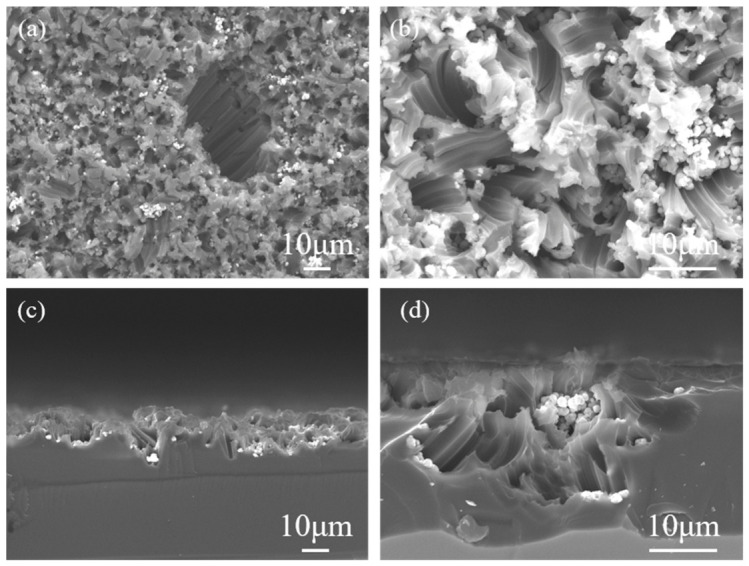
Top-view SEM images (**a**,**b**) and cross-sectional SEM images (**c**,**d**) of RuO_2_-2-coated p-Si (100) 15–25 Ω·cm substrate etched in 0.4 M H_2_O_2_-10 M HF solution for 1 h.

Figure 6 illustrates the etching behavior of RuO_2_-2-coated Si under identical conditions. Similarly, etching occurs only at sites where RuO_2_-2 particles were present. The spherical RuO_2_-2 particles (Figure 6a,b) induced disordered surface etching with tilted or rotated pores. This phenomenon arises from the mobility and aggregation of small particles in solution, as evidenced by clustered RuO_2_-2 particles (Figure 6b). Cross-sectional SEM (Figure 6c,d) reveals pore depths of 10–30 µm. At sites with fewer RuO_2_-2 particles, the catalytic activity was weaker and the etching reaction proceeded more slowly. Excessive accumulation of RuO_2_-2 particle hindered the access of the solution to the reaction interface, suppressing the oxidation and dissolution of silicon and resulting in relatively shallow etching depths. Therefore, the density of RuO_2_-2 particles on the silicon surface plays a critical role in the etching process. The etching behavior of Si is known to be crystallographically anisotropic, with etching rates varying across different orientations due to differences in surface bond density and reactivity. According to the principle of minimum energy, preferential etching is expected to proceed along the <100> direction. However, our observations suggest that the mobility of RuO_2_ particles during the etching process introduces additional complexity. For isolated or sparsely distributed RuO_2_ particles, the etching direction can change dynamically, following the moving trajectory of the catalyst particles. In contrast, in regions where RuO_2_ particles are aggregated, their mobility is spatially constrained, and the etching proceeds predominantly along the <100> direction. Therefore, the final pore morphology is determined by the interplay between Si crystallographic anisotropy and the local distribution and mobility of the RuO_2_ catalyst.

**Figure 7:**
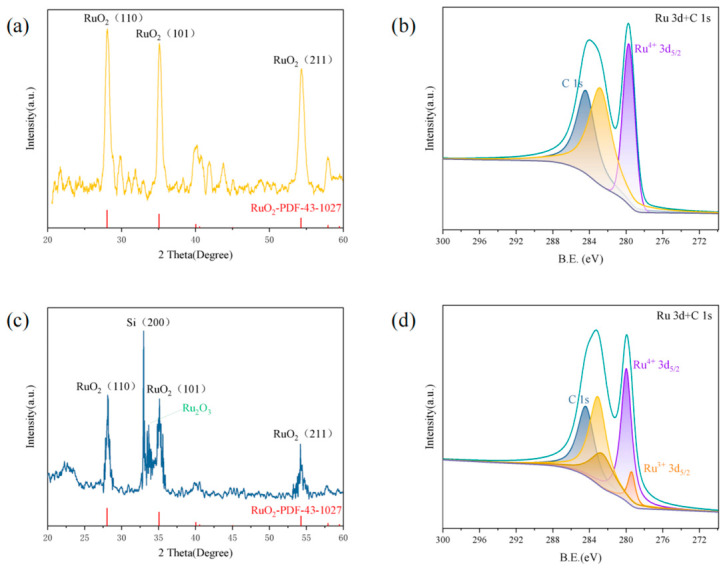
XRD pattern of (**a**) RuO_2_ and (**c**) RuO_2_-coated p-Si (100) 15–25 Ω·cm substrate etched in 0.4 M H_2_O_2_-10 M HF solution for 1 h; Deconvoluted XPS spectra of Ru 3d+C 1s of (**b**) RuO_2_ and (**d**) RuO_2_-coated p-Si (100) 15–25 Ω·cm substrate etched in 0.4 M H_2_O_2_-10 M HF solution for 1 h.

To determine whether the catalyst undergoes chemical transformation during etching, RuO_2_ before and after catalyzing the silicon etching were analyzed by XRD (Figure 7a,c) and XPS (Figure 7b,d). The characteristic peaks at 28.08°, 35.10°, and 54.32°, corresponding to (110), (101), and (211) crystal planes of rutile RuO_2_, respectively, are consistent with the RuO_2_ phase (JCPDS #43-1027) [33]. The XRD patterns of etched silicon wafers coated with RuO_2_ exhibit two additional peaks besides the characteristic peaks of RuO_2_. The characteristic peaks at 32.98° and 33.66° correspond to the (200) crystal plane of single-crystal silicon and the characteristic peak of Ru_2_O_3_, respectively, indicating the formation of Ru_2_O_3_. To ensure reliable phase identification, the standard peak positions of Ru_2_O_3_ [30,34] are provided in the Supporting Information (Figure S1) for direct comparison with the experimental XRD pattern. Figure 7b,d show the XPS spectra in the Ru 3d region. The Ru 3d core-level spectrum was deconvoluted using a mixed Gaussian-Lorentzian function after Shirley background subtraction. Before catalyzing the etching of silicon, only one Ru 3d spin–orbit doublet was observed. The component at 279.9 eV (Ru 3d_5/2_) is attributed to Ru^4+^, which is consistent with the peak position of RuO_2_ (280.3 eV) [35]. The slight shift in the peak position can be ascribed to the RuO_2_ particles being in an electron-rich state. The Ru^3+^ 3d_5/2_ peak is identified at 279.1 eV, which is exactly 0.8 eV lower than the Ru^4+^ peak. This separation is highly consistent with the work of Sayan et al. [36], where the Ru^3+^ 3d_5/2_ peak is typically found approximately 0.8 eV below that of Ru^4+^.

**Figure 8:**
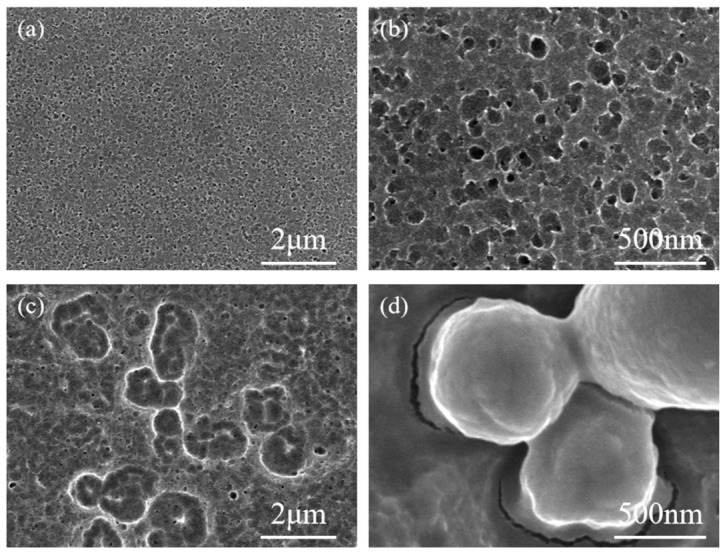
(**a**,**b**) Top-view SEM images of RuO_2_-coated p-Si (100) 15–25 Ω·cm substrate etched in the aerated HF/H_2_O vapor for 5 h; (**c**,**d**) Top-view SEM images of RuO_2_-coated p-Si (100) 15–25 Ω·cm substrate etched in the aerated HF/H_2_O vapor for 22 h.

To further investigate the role of the oxidant in the etching process, we replaced H_2_O_2_ with O_2_ and compared the resulting etching behavior. The RuO_2_-coated silicon wafers were transferred into a PTFE reactor containing aerated HF/H_2_O vapor. Figure 8 shows the SEM images of wafers etched in aerated HF/H_2_O vapor at 295 K for different durations. After 5 h, only a porous layer formed on the surface, with no evidence of deep pore propagation. When the etching time was extended to 22 h, RuO_2_ particles remained embedded on the silicon surface without further downward etching. In contrast, deep pores (up to 60 μm) were readily formed within 1 h (Figure 2) when H_2_O_2_ was used as the oxidant.

**Figure 9:**
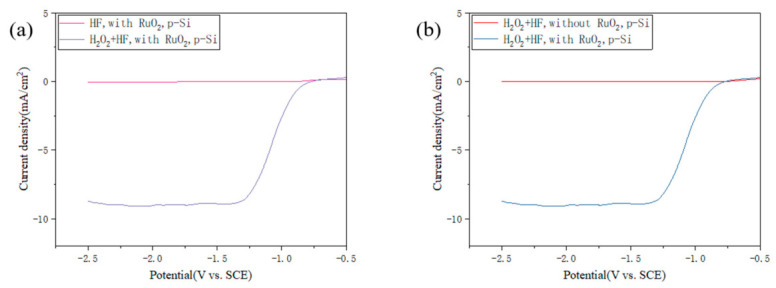
(**a**) Dark cathodic polarization curves of RuO_2_-plated silicon wafers in HF (pink curve)/HF-H_2_O_2_ (purple curve) solution; (**b**) Dark cathodic polarization curves of silicon wafers in HF-H_2_O_2_ solution without RuO_2_ (red curve) and with RuO_2_ (blue curve).

The oxidation of Ru_2_O_3_ to RuO_2_ by H_2_O_2_ (Reaction (2)) is known to be both thermodynamically favorable and kinetically fast under acidic conditions, as H_2_O_2_ is a strong two-electron oxidant with a standard reduction potential of +1.78 V (H_2_O_2_ + 2H^+^ + 2e^−^ → 2H_2_O). However, the oxidation of Ru_2_O_3_ by molecular oxygen (O_2_ + 4H^+^ + 4e^−^ → 2H_2_O, E° = +1.23 V) is thermodynamically less favorable and, more importantly, exhibits extremely slow kinetics at room temperature. In the dark state, the magnitude of the cathodic polarization current reflects the strength of hole injection. The greater the polarization current, the stronger the ability to inject holes into the valence band of silicon. We compared the cathodic polarization curves of RuO_2_-plated silicon wafers in HF solution with H_2_O_2_ or dissolved O_2_ (Figure 9a). The polarization current of the RuO_2_-plated silicon wafer in HF solution with dissolved O_2_ was an order of magnitude smaller than that of the RuO_2_-plated silicon wafer in HF-H_2_O_2_ solution. While RuO_2_ can still inject holes into silicon and be reduced to Ru_2_O_3_ at the Si/RuO_2_ interface, the reoxidation of Ru_2_O_3_ back to RuO_2_ by O_2_ is inefficient. The accumulation of Ru_2_O_3_ at the interface rapidly suppresses further hole injection, leading to a self-limiting behavior in which only shallow surface etching occurs and small cathode current is observed. In contrast, H_2_O_2_ can efficiently reoxidize Ru_2_O_3_ to RuO_2_, sustaining the cyclic redox process and hole injection into the valence band of silicon, resulting in the generation of a large cathodic current and continuous deep pore formation. The dark cathodic polarization current of the RuO_2_-plated silicon wafer was significantly greater than that of the pure silicon wafer in HF-H_2_O_2_ solution (Figure 9b), indicating that the presence of RuO_2_ greatly enhances the ability of hole injection.

**Figure 10:**
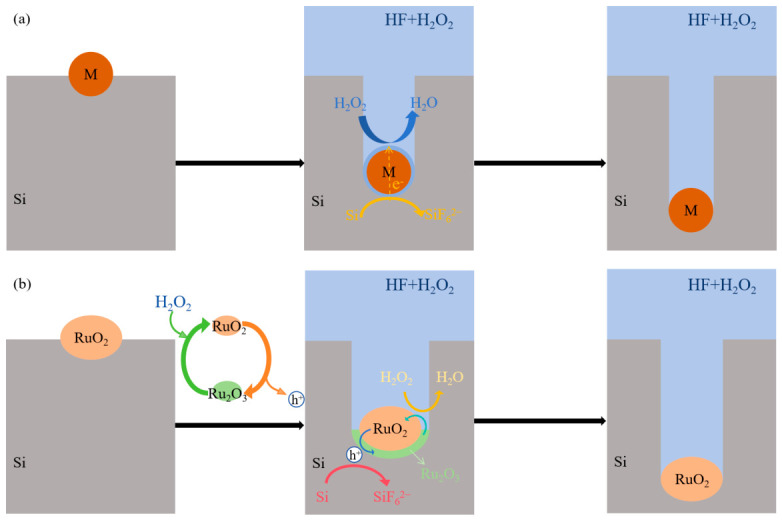
Schematic diagrams of (**a**) MACE mechanism and (**b**) RuO_2_-catalyzed silicon etching mechanism.

The schematic illustration of the MACE mechanism is shown in Figure 10a. In conventional MACE, the noble metal catalyst (such as Au, Ag, or Pt) remains chemically unchanged throughout the etching process. It serves as a cathodic site that confines the etching reaction to localized sites, attracting electrons from silicon. The oxidant (such as H_2_O_2_) extracts electrons from silicon at the metal particle surface and injects holes into silicon, thereby enabling rapid etching [26,27,28]. In the RuO_2_-catalyzed silicon etching process, we suggest that RuO_2_ particles serve two functions in catalyzing silicon etching: (1) confining the silicon etching reaction to the Si/ RuO_2_ interface, and (2) injecting holes into the valence band of silicon to initiate oxidation. A schematic illustration of the RuO_2_-catalyzed silicon etching mechanism is shown in Figure 10b. At the Si/ RuO_2_ interface, RuO_2_ particles in contact with silicon are reduced to Ru_2_O_3_ by extracting electrons from the silicon valence band, as described by Reaction (1). Since the etchant contains H_2_O_2_, a strong oxidizing agent, Ru_2_O_3_ is rapidly oxidized by H_2_O_2_ back to RuO_2_ via Reaction (2). It should be noted that ruthenium species predominantly exist as RuO_2_ throughout the process, with the generated intermediate species (Ru_2_O_3_) being transient and localized around the RuO_2_ particles. The ruthenium species undergo the cyclic redox process until the hydrogen peroxide is exhausted. Substantial holes accumulate at the Si/ RuO_2_ interface, initiating anodic oxidation of silicon through both four-electron (Reactions (3) and (4)) and two-electron (Reaction (5)) pathways. This electrochemical process generates hydrogen gas and yields water-soluble hexafluorosilicic acid (H_2_SiF_6_) as the primary reaction product. The overall reaction related to silicon can be comprehensively described by Reaction (6). Table 1 shows a comparison between MACE and OACE in terms of charge transfer, catalyst behavior, and reaction pathways.

(1) $$2\mathrm{Ru}O_{2}+2H^{+}+2e^{-}\to {\mathrm{Ru}}_{2}O_{3}+{\;H}_{2}O\;\;\;E=0.973V$$

(2) $${\mathrm{Ru}}_{2}O_{3}+{\;H}_{2}O_{2}\to 2\mathrm{Ru}O_{2}+H_{2}O$$

(3) $$\mathrm{Si}+2H_{2}O+4h^{+}\to \mathrm{Si}O_{2}+4H^{+}\;\;\;E=-0.84V$$

(4) $$\mathrm{Si}O_{2}+6\mathrm{HF}\to H_{2}\mathrm{Si}F_{6}+2H_{2}O$$

(5) $$\mathrm{Si}+6F^{-}+2H^{+}+2h^{+}\to \mathrm{Si}F_{6}^{2-}+H_{2}\;\;\;E=-1.2V$$

(6) $$\mathrm{Si}+6\mathrm{HF}+\frac{n}{2}H_{2}O_{2}\to H_{2}\mathrm{Si}F_{6}+nH_{2}O+\left(\frac{4-n}{2}\right)H_{2}\;$$

## 4. Conclusions

In summary, this work elucidates the fundamental mechanism of oxide-catalyzed silicon etching in oxidizing HF solutions. Morphological characterization and compositional analysis demonstrated that Ru_2_O_3_ is generated during the etching process of RuO_2_-coated silicon in HF-H_2_O_2_ solutions. RuO_2_ injects holes into the valence band of silicon, thereby initiating silicon oxidation. Hydrogen peroxide serves to oxidize Ru_2_O_3_, sustaining the oxidation reaction of silicon. The ruthenium species undergo a cyclic process involving reduction of RuO_2_ to Ru_2_O_3_ and subsequent reoxidation to RuO_2_ by H_2_O_2_. The oxidized silicon is dissolved by hydrofluoric acid, ultimately resulting in the formation of deep pores. The proposed mechanism provides a complementary perspective to the existing models of OACE.

## Figures and Tables

**Table 1:** Comparison between MACE and OACE.

	MACE (Noble Metals: Ag, Au, Pt, etc.)	OACE (This Work: RuO_2_)
Charge transfer	Oxidant (e.g., H_2_O_2_) injects holes into silicon.	RuO_2_ particles inject holes into silicon.
Catalyst behavior	The valence state of the catalyst remains unchanged; Metal particles move directionally.	The valence state of the catalyst changes; RuO_2_ particles move directionally.
Reaction pathways	The oxidant (e.g., H_2_O_2_) injects holes into silicon through the catalyst, and the oxidized silicon dissolves in the HF solution.	RuO_2_ is reduced to Ru_2_O_3_ after injecting holes into silicon, and the oxidized silicon dissolves in the HF solution. Ru_2_O_3_ is reoxidized to RuO_2_ by H_2_O_2_.

## Data Availability

The original contributions presented in this study are included in the article/Supplementary Materials. Further inquiries can be directed to the corresponding author.

## Supplementary Materials

The following supporting information can be downloaded at: https://www.mdpi.com/article/10.3390/ma19091734/s1, Figure S1: XRD patterns of Ru_2_O_3_ and RuO_2_.
