# Supplementary material for: The Mechanism of Ruthenium Oxide Catalyzed Electroless Etching of Silicon in Oxidizing HF Solution
Source: Materials (Basel). 2026 Apr 24;19(9):1734. doi: 10.3390/ma19091734 (PMC13164636; doi:10.3390/ma19091734)
Supplement: Supplementary file 1 [file materials-19-01734-s001.zip › materials-4246799-supplementary.pdf]

Supporting Information

RuO_2 catalyze the corrosion of silicon in HF/H_2O_2 solution

Bing Bai, Yingqi Li, Wei Xu, Peiao Lu, Jiakun Luo, Jinyu Wu, Kui-Qing Peng*

Key Laboratory of Multiscale Spin Physics, Ministry of Education, Beijing Key Laboratory of Energy Conversion and Storage Materials, School of Physics and Astronomy, Beijing Normal University, Beijing, 100875, PR China
*Corresponding author.
E-mail: kq_peng@bnu.edu.cn

Supporting Materials:

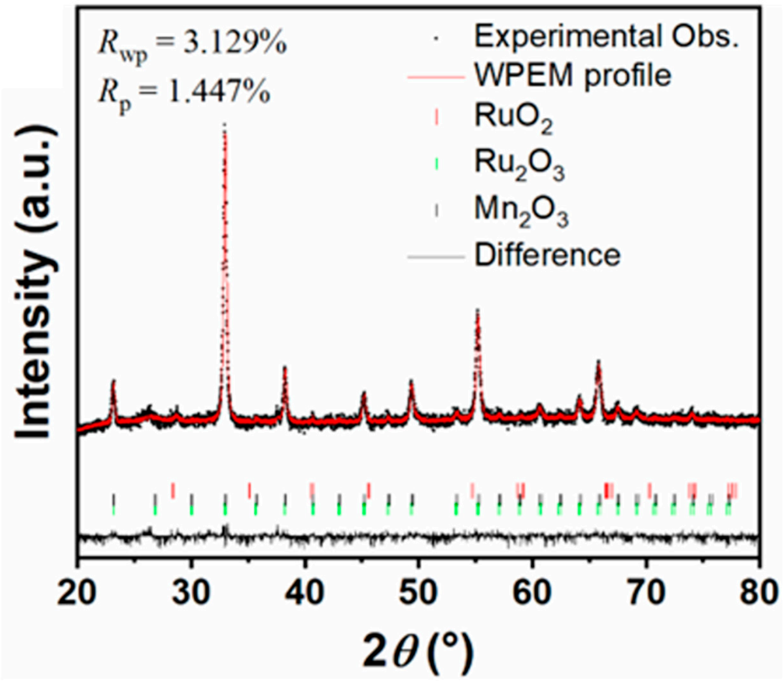

Figure S1: XRD patterns of Ru_2O_3 and RuO_2.
